# Duck TRIM29 negatively regulates type I IFN production by targeting MAVS

**DOI:** 10.3389/fimmu.2022.1016214

**Published:** 2023-01-06

**Authors:** Weiqiang Li, Yating Song, Yuqing Du, Zhanhong Huang, Meng Zhang, Zuxian Chen, Zhuoliang He, Yangbao Ding, Junsheng Zhang, Luxiang Zhao, Hailiang Sun, Peirong Jiao

**Affiliations:** ^1^ Guangdong Laboratory for Lingnan Modern Agriculture, College of Veterinary Medicine, South China Agricultural University, Guangzhou, China; ^2^ Key Laboratory of Animal Vaccine Development, Ministry of Agriculture and Rural Affairs, Guangzhou, China; ^3^ Guangdong Provincial Key Laboratory of Zoonosis Prevention and Control, Guangzhou, China

**Keywords:** duck, TRIM29, Type I IFN, MAVS, innate immune response

## Abstract

The innate immune response is a host defense mechanism that induces type I interferon and proinflammatory cytokines. Tripartite motif (TRIM) family proteins have recently emerged as pivotal regulators of type I interferon production in mammals. Here, we first identified duck TRIM29, which encodes 571 amino acids and shows high sequence homology with other bird TRIM29 proteins. DuTRIM29 inhibited IFN-β and IRF7 promoter activation in a dose-dependent manner and downregulated the mRNA expression of IFN-β, IRF7, Mx and IL-6 mediated by duRIG-I. Moreover, duTRIM29 interacted and colocalized with duMAVS in the cytoplasm. DuTRIM29 interacted with duMAVS *via* its C-terminal domains. In addition, duTRIM29 inhibited IFN-β and IRF7 promoter activation and significantly downregulated IFN-β and immune-related gene expression mediated by duMAVS in ducks. Furthermore, duTRIM29 induced K29-linked polyubiquitination and degradation of duMAVS to suppress the expression of IFN-β. Overall, our results demonstrate that duTRIM29 negatively regulates type I IFN production by targeting duMAVS in ducks. This study will contribute to a better understanding of the molecular mechanism regulating the innate immune response by TRIM proteins in ducks.

## Introduction

Innate immunity acts as the first line of defense against pathogen invasion. Innate immune responses are activated by pattern-recognition receptors (PRRs), which recognize conserved pathogen-associated molecular patterns (PAMPs) (1). Retinoic acid-inducible gene I-like receptors (RLRs), as PRRs, have been identified to recognize nonself nucleic acids. RLRs include three members: RIG-I, MDA5 and LGP2. RIG-I was identified as the first RLR, which mainly recognizes cytoplasmic 5’ppp dsRNA and short-chain dsRNA (2). Upon activation by PAMPs, RIG-I recruits downstream adaptor proteins such as MAVS, TRAF3 and TBK1 to trigger the activation of the transcription factors IRF3, IRF7 and NF-κB, which causes IFN-I and proinflammatory cytokine production (3).

Tripartite motif (TRIM) proteins are a family of over 80 members in human and possess a conserved RBBC motif (4). In ducks, several TRIM proteins have identified including TRIM25, TRIM27 and TRIM32 (5–7). TRIM29, also named as ATDC, is a member of TRIM family. As a multifunctional protein, emerging evidence have indicated that TRIM29 plays a crucial role in regulating innate immunity, antiviral function and tumor formation in mammals (8–12). However, TRIM29 whether exists in ducks and its functions in innate immunity are currently unknown.

The RIG-I signaling pathway is precisely regulated by multiple posttranslational modifications including ubiquitination, phosphorylation, sumoylation and acetylation (13, 14). Ubiquitination modification plays an essential role in changing protein function and degradation mediated by the proteasome system. TRIM proteins are an E3 ubiquitin ligase family, and several TRIM proteins have been reported to modulate the RIG-I pathway *via* ubiquitination. For example, TRIM25 acts as a positive regulator by mediating the ubiquitination of RIG-I and MAVS (15, 16). TRIM35 regulates innate immune responses by catalyzing the ubiquitination of TRAF3 and IRF7 (17, 18). These studies indicate that TRIM proteins regulating the RIG-I signaling pathway mediate innate immune responses by targeting key proteins. In mammals, TRIM29 induced polyubiquitination and proteasomal degradation of NEMO, MAVS and STING, thereby inhibiting the expression of IFN-I (8–11). However, the role of duTRIM29 in RIG-I signaling pathway mediated IFN-I production in ducks remains unclear.

Here, we cloned the duck TRIM29 (named duTRIM29) gene from duck embryo fibroblast cells and explored its function in the IFN-I signaling pathway in ducks. Additionally, duTRIM29 inhibited IFN-β and immune-related gene expression by targeting duMAVS. Thus, we identified duTRIM29 as an important negative regulator of IFN-β production in the RIG-I signaling pathway in ducks.

## Materials and methods

### Cell lines and reagents

HEK293T cells and shelduck embryo fibroblast (DEF) cells were maintained at 37°C in DMEM (Gibco), enriched with 10% FBS (Gibco). Anti-HA (26183), anti-MYC (MA1-980), anti-FLAG (MA1-91878) and anti-GAPDH (MA515738) antibodies were purchased from Invitrogen. Anti-HA agarose beads (26182) and anti-V5 agarose beads (A7345) were purchased from Invitrogen and Sigma, respectively. Low molecular weight (LMW)-poly(I:C) and anti-V5 (ab27671) antibody were obtained from Invivogen and Abcam, respectively. IRDye^®^ 800CW goat anti-rabbit IgG (C50331-03) and IRDye^®^ 800CW goat anti-mouse IgG (C41028-02) secondary antibodies were from LI-COR. DMSO, MG132, bortezomib, bafilomycin A1 (Baf A1), chloroquine (CQ) and 3-Methyladenine (3-MA) were purchased from MedChemExpress.

### Cloning and bioinformatic analysis of duTRIM29

Specific primers (duTRIM29-F/R) were designed according to the predicted sequence of duck TRIM29 in GenBank (accession number: XM_027444322.2) (**Table 1**). The complete CDS of duTRIM29 was amplified using cDNA synthesized with total RNA by RT-PCR. The PCR products were sequenced to obtain the duTRIM29 sequence. The nucleotide sequence homology between duck and other species was compared by MegAlign. The amino acid sequences of TRIM29 were aligned by ClustalX. The phylogenetic tree of TRIM29 was built using MEGA 5.0. The domains of duTRIM29 were predicted by SMART (http://smart.embl-heidelberg.de/).

**Table 1:** Primer sequences used in this study.

Primer name	Primer sequence (5’-3’)	Purpose
duTRIM29-FL-F	CGCGGGCAGGCACGCGGACGGA	Full length gene cloning
duTRIM29-FL-R	CCCTCTCCCTGGCAGAGATT	
duTRIM29-F	GACGATGGAAACGGGGAGCGCAGCAA	Gene cloning
duTRIM29-R	GGAATCAAGGTGCCTCGTTGGAGG	
duTRIM29-HA-F	CGAGAATTCATGTACCCATACGACGTCCCAGACTACGCTGAAACGGGGAGCGCAGCAAGG	Eukaryotic expression plasmids construction
duTRIM29-HA-R	CTACTCGAGTCAAGGTGCCTCGTTGGAGGTGG	
duTRIM29(241-571 aa)-HA-F	CGAGAATTCATGTACCCATACGACGTCCCAGACTACGCTGCAAGAAAATGCCCT	
duTRIM29(241-571 aa)-HA-R	CTACTCGAGTCAAGGTGCCTCGTTGGAGGTGG	
duTRIM29(278-571 aa)-HA-F	CAGAATTCATGTACCCATACGACGTCCCAGACTACGCTACGGTGGAGATCGAGAAAG	
duTRIM29(278-571 aa)-HA-R	CTACTCGAGTCAAGGTGCCTCGTTGGAGGTGGAGGT	
duTRIM29(369-571 aa)-HA-F	CGAGAATTCATGTACCCATACGACGTCCCAGACTACGCTCTGGAAGAGAGGGCG	
duTRIM29(369-571 aa)-HA-R	CTACTCGAGTCAAGGTGCCTCGTTGGAGGTGGAGGT	
duTRIM29(1-368 aa)-HA-F	CGAGAATTCATGTACCCATACGACGTCCCAGACTACGCTGAAACGGGGAGCGCAG	
duTRIM29(1-368 aa)-HA-R	CTACTCGAGTCACTCATCCACAATCTCCT	
duTRIM29-AsRed-F1	CGAGAATTCATGGCCTCTTTGCTGAAGA	
duTRIM29-AsRed-R1	CGATCCGCCACCGCCAGAGCCACCTCCGCCTGAACCGCCTCCACCGTTGTGGCCCAGCTTGGAGGGG	
duTRIM29-AsRed-F2	GGTGGAGGCGGTTCAGGCGGAGGTGGCTCTGGCGGTGGCGGATCGGAAACGGGGAGCGCAGCAAGGA	
duTRIM29-AsRed-R2	TACTCGAGTCAAGGTGCCTCGTTGGAGG	
q-duIFN-β-F	CAGCATCAACAAGTACTTCA	qRT-PCR
q-duIFN-β-R	CTTCCGAAGTGGCTGGGAGA	
q-duIRF7-F	CCACACCTGGATGTCACCAT	qRT-PCR
q-duIRF7-R	AGACGTGCTGCCCCGGCTGC	
q-duMx-F	CCAGACCTGACACTAATTGAT	qRT-PCR
q-duMx-R	CACATTACATGGCACCACTAC	
q-duIL 6-F	CAGACCTACCTTGAATACGTA	qRT-PCR
q-duIL 6-R	AGCTGAATCTGGGATGACCAC	
q-duPKR-F	GGAAGCAAGAGCAGTAGCAGC	qRT-PCR
q-duPKR-R	GTACTCATTTAGTAGACTGAC	
q-duOAS-F	GGAGCTGTTGACCATCTATAC	qRT-PCR
q-duOAS-R	CGATCCGGTGATGCTGCAGCG	
q-duβ-actin-F	GATCACAGCCCTGGCACC	qRT-PCR
q-duβ-actin-R	CGGATTCATCATACTCCTGCTT	
q-duTRIM29-F	GAAGGAGATTGTGGATGAGCTGGAAG	qRT-PCR
q-duTRIM29-R	TGGAGGAAGAGCACGGAGTCAC	

### Construction and transfection of plasmids

The full-length duTRIM29 with N-terminal HA tag, duTRIM29 with N-terminal AsRed tag and truncation mutants of duTRIM29 with N-terminal HA-tag were amplified and then subcloned into the pCAGGS vector. All the primers for eukaryotic expression plasmid construction are listed in **Table 1**. The expression plasmids of full-length duRIG-I with C-terminal HA tag, full-length duRIG-I with C-terminal MYC tag, full-length duMAVS and TBK1 with N-terminal V5 tag, full-length duIRF7 with N-terminal FLAG tag and full-length Ub with N-terminal MYC tag were constructed and stored in our lab. pGL3-IFN-β-Luc and pGL3-IRF7-Luc plasmids were constructed as previously described (19). Ub-WT-HA, Ub-K6-HA, Ub-K11-HA, Ub-K27-HA, Ub-K29-HA, Ub-K33-HA, Ub-K48-HA and Ub-K63-HA were obtained from Addgene. The plasmids were transfected into HEK293T cells and DEF cells *via* Lipofectamine 2000 (Invitrogen) following the manufacturer’s instructions.

### RNA interference

Three small interfering RNAs (siRNAs) targeting duTRIM29 and negative control (siNC) were synthesized by RiboBio company (Guangzhou, China). The sequences of three siRNAs are listed in **Table 2**. DEF cells were plated on 6-well plates, 50 nM siRNA and NC were transfected into DEF cells. The interference efficiency was analyzed by qRT-PCR after 24h of transfection. In addition, HEK293T cells were plated on 6-well plates, duTRIM29-HA was transfected into HEK293T cells. After 12 h, 50 nM siRNA and NC were transfected into HEK293T cells. The interference efficiency was assessed by detecting the protein expression of duTRIM29 after 24h of transfection. According to the interference efficiency of TRIM29 in mRNA and protein levels, we selected a siRNA with better interference efficiency for the follow-up experiments.

**Table 2:** Sequences of small interfering RNA.

Name	Sense sequence (5’-3’)	Antisense sequence (5’-3’)
siTRIM29-1	GCUGAAGAUCAUCGAGGUATT	UACCUCGAUGAUCUUCAGCTT
siTRIM29-2	CAGUAGACCAGCAUUUCAATT	UUGAAAUGCUGGUCUACUGTT
siTRIM29-3	ACCAGAUGUGCAUCUGCUATT	UAGCAGAUGCACAUCUGGUTT

### Luciferase reporter gene assays

DEF cells were plated on 24-well plates. After 24 h, 300 ng or 500 ng of duTRIM29-HA, 300 ng duRIG-I-MYC and 300 ng duMAVS-V5 together with 100 ng pGL3-IFNβ-Luc or 100 ng pGL3-IRF7-Luc and 10 ng pTK-RL were transfected into DEF cells. The amount of plasmid transfected per well was made up to 1.5 μg using the empty vector pCAGGS. After 24 h, the luciferase activities of the total cell lysates were measured by the Dual-Luciferase Reporter Assay System (Promega) according to the manufacturer’s protocol. Luciferase activity was measured by a Promega Glomax 96 microplate luminometer. The relative luciferase activity was calculated by the ratio of firefly to Renilla luciferase activity as previously described (20). All reporter assays were repeated three times independently and three replicate wells were done in each assay.

### Quantitative RT-PCR

DEF cells were plated on 6-well plates. After 24 h, 2 μg duTRIM29-HA, 2 μg du-RIG-I-MYC and 2 μg duMAVS-V5 were transfected into DEF cells. The amount of plasmid transfected per well was made up to 4 μg using the empty vector pCAGGS. After another 24 h, DEF cells were lysed and total RNA was extracted using RNAfast200 reagent (Fastagen). Total RNA was quantified and used to synthesize cDNA with a Reverse Transcription System (Promega). qRT-PCR was performed with specific primers on a CFX96 Real-Time PCR machine (Bio-Rad). The primers for qRT-PCR are listed in **Table 1**. The relative fold induction was analyzed by the 2^−ΔΔCt^ method as previously described (21). All qRT-PCR assays were repeated three times.

### Co-immunoprecipitation and immunoblot analysis 

HEK293T cells were plated on 100 mm dishes and cultured for 24 h. Then, duTRIM29-HA and du-RIG-I-MYC, duMAVS-V5, duTBK1-V5 or duIRF7-FLAG expression plasmids were cotransfected into HEK293T cells. At 24 h posttransfection, HEK293T cells were lysed with 600 μl NP-40 lysis buffer (Beyotime) containing 1% PMSF. Eighty microliters of lysate was used as input to detect whether proteins were expressed, and the remaining lysate was incubated with anti-V5 or anti-HA agarose beads for 4-6 h at 4°C to detect the protein-protein interaction. The proteins of the input and beads were separated by SDS-PAGE, and the bands of the PAGE gel were transferred to the NC membrane. Then, the membrane was blocked with 5% BSA and incubated with primary antibody overnight at 4°C. Finally, the NC membrane was incubated with the secondary antibody for 1 hour. The proteins in the NC membrane were detected by a Dual Color Infrared Laser Imaging System (LI-COR).

### Confocal microscopy

HEK293T cells and DEF cells were grown on sterilized coverslips in 24-well plates overnight. After 24 h, duMAVS-eGFP and duTRIM29-AsRed were cotransfected into HEK293T cells and DEF cells, respectively. The cells on the coverslips were settled in 4% cell fixative for 15 min. Then, the cells were permeabilized with 0.1% Triton X-100 for 10 min and mounted with ProLong™ Gold Antifade Mountant (Invitrogen). The nuclear DNA of cells was stained with DAPI (Invitrogen) for 10 min. The subcellular localization of duMAVS-eGFP and duTRIM29-AsRed was observed by TCS SP8 confocal microscopy (Leica).

### Statistical analysis

The data are presented as the mean ± standard deviation and the statistical analyses were performed by GraphPad Prism 7. Differences between two groups were analyzed by unpaired two-tailed Student’s t test, and P<0.05 was considered statistically significant.

## Results

### Cloning and sequence analysis of duTRIM29

**Figure 1:**
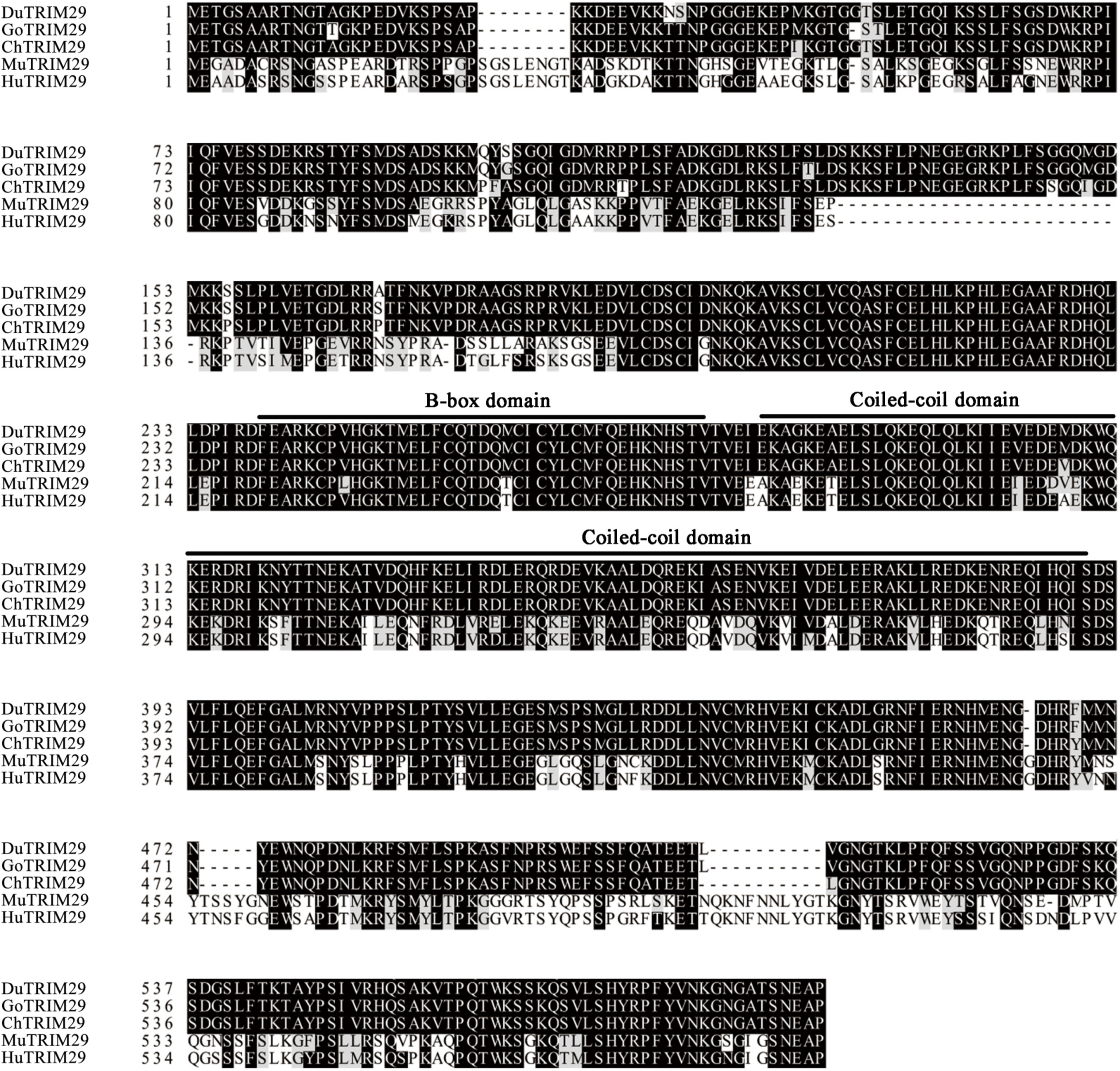
Amino acid sequence alignment of duck TRIM29 with goose, chicken, mouse and human TRIM29. The amino acid sequences of TRIM29 were aligned by ClustalX and edited by BOXSHADE. Accession numbers: human (NP_036233.2), mouse (NP_076144.2), chicken (XP_417892.4) and goose (XP_013042747.1). TRIM29 sequences are shown for duck (Du), goose (Go), chicken (Ch), mouse (Mu) and human (Hu).

**Figure 2:**
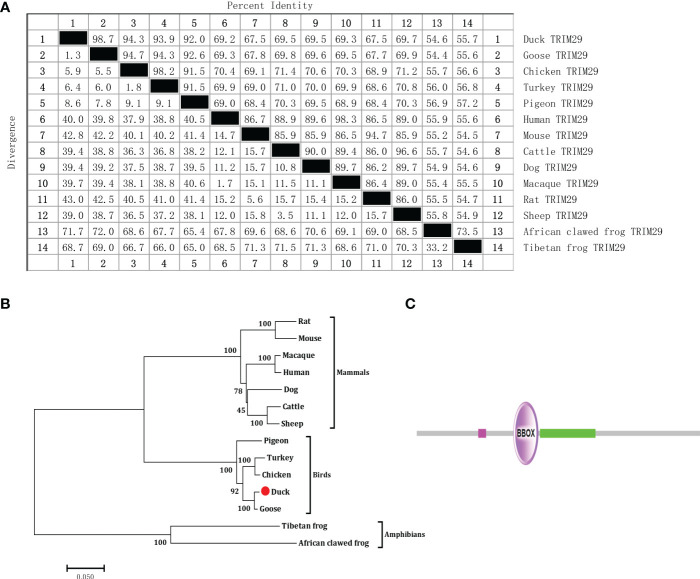
Nucleotide sequence homology, phylogenetic tree of TRIM29 between duck and other species and protein domains of duTRIM29. **(A)** Nucleotide sequence homology comparison of TRIM29 between duck and other vertebrates. **(B)** Phylogenetic tree of TRIM29. The phylogenetic tree was built by MEGA 5.0 based on the amino acid sequence of TRIM29 from NCBI. Accession numbers: goose (XP_013042747.1); cattle (XP_024831057.1); dog (XP_038520248.1); pigeon (XP_021140921.1); chicken (XP_417892.4); human (NP_036233.2); macaque (NP_001244975.1); turkey (XP_010721967.1); mouse (NP_076144.2); tibetan frog (XP_018414950.1); sheep (XP_027835126.2); rat (NP_001100285.1); and african clawed frog (XP_004916176.1). **(C)** Predicted functional domain map of duTRIM29. Conserved functional domains were analyzed using the amino acid sequence of duck TRIM29 by SMART software. The domains are shown in purple and green. Low-complexity regions are shown in fuchsia.

To identify duck TRIM29, full-length duTRIM29 cDNA was amplified from duck embryo fibroblast cells using primers (duTRIM29-F/R) by RT-PCR. As shown in **Figure 1**, the open reading frame of duTRIM29 contained 1716 bp and encoded a protein of 571 amino acids (GenBank accession number: OP822045) (**Figure 1**). At the nucleotide sequence level, duTRIM29 was highly homologous with goose, chicken and other birds (89.3%-98.6%), moderately homologous with human, mouse and other mammals (62.2%-64.7%), and weakly homologous with tibetan frog (46.4%) and african clawed frog (44.6%) (**Figure 2A**). The phylogenetic analysis of TRIM29 showed that duck, goose, chicken, turkey and pigeon TRIM29 belong to the bird clade (**Figure 2B**). The results of domain prediction indicated that duTRIM29 included a B-box domain (amino acids 241-277) and a coiled-coil domain (amino acids 278-368) (**Figure 2C**).

### DuTRIM29 inhibits duRIG-I-mediated IFN-β production

**Figure 3:**
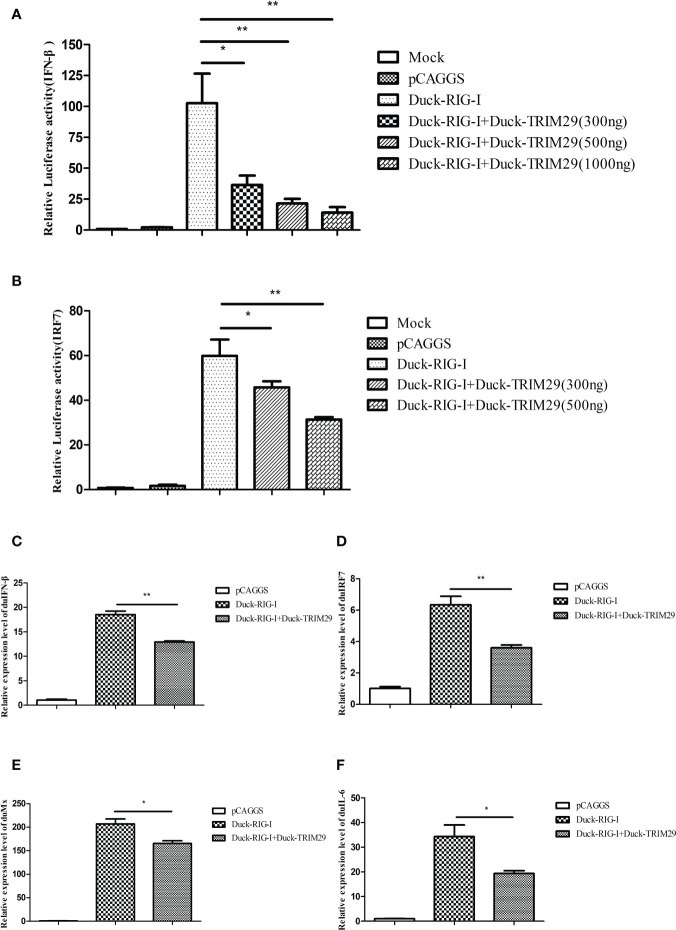
Overexpression of duTRIM29 inhibits duRIG-I-mediated IFN-β and immune-related gene expression. **(A, B)** DEF cells were cotransfected with duTRIM29-HA, duRIG-I-MYC, or pCAGGS together with IFN-β-Luc**(A)**, IRF7-Luc**(B)** and pRL-TK. After 24 h, the cells of the duTRIM29-HA transfection group and the duTRIM29-HA and duRIG-I-MYC cotransfection group were stimulated with 5 μg/ml poly (I:C). IFN-β and IRF7 promoter activity were measured after 12 h. **(C–F)** DEF cells were cotransfected with duRIG-I-MYC, duTRIM29-HA, or pCAGGS. After 24 h, the cells of the duTRIM29-HA transfection group and the duTRIM29-HA and duRIG-I-MYC cotransfection group were stimulated with 5 μg/ml poly (I:C). After 12 h, the mRNA levels of IFN-β **(C)**, IRF7 **(D)**, Mx **(E)** and IL-6 **(F)** were measured by qRT-PCR. Statistics were analyzed *via* unpaired two-tailed Student’s t test: *P < 0.05; **P < 0.01.

**Figure 4:**
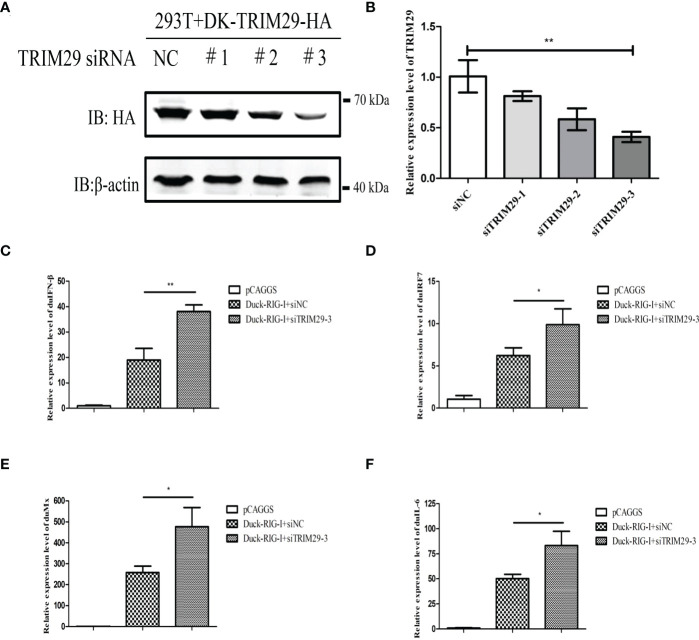
Knockdown of duTRIM29 upregulates duRIG-I-mediated IFN-β production. **(A)** HEK293T cells were plated on 6-well plates, duTRIM29-HA was transfected into HEK293T cells. After 12 h, 50 nM siRNA and NC were transfected into HEK293T cells. The interference efficiency was assessed by detecting the protein expression of duTRIM29 after 24h of transfection. **(B)** DEF cells were plated on 6-well plates, 50 nM siRNA and NC were transfected into DEF cells. The interference efficiency was analyzed by qRT-PCR after 24h of transfection. **(C–F)** DEF cells were cotransfected with duRIG-I-MYC, siTRIM29-3, or siNC. After 24 h, the cells of the siNC and duRIG-I-MYC cotransfection group and the siTRIM29-3 and duRIG-I-MYC cotransfection group were stimulated with 5 μg/ml poly (I:C). After 12 h, the mRNA levels of IFN-β**(C)**, IRF7**(D)**, Mx**(E)** and IL-6**(F)** were measured by qRT-PCR. Statistics were analyzed *via* unpaired two-tailed Student’s t test: *P < 0.05; **P < 0.01.

To investigate the role of duTRIM29 in the duRIG-I-mediated type I IFN signaling pathway, we examined the expression of IFN-β and immune-related gene after overexpression or knockdown of duTRIM29 and duRIG-I in DEF cells. The results showed that duTRIM29 potently inhibited avian IFN-β and IRF7 promoter activation in a dose-dependent manner (**Figure 3**). qRT-PCR results indicated that duTRIM29 significantly downregulated the expression of IFN-β, IRF7, Mx and IL-6 (**Figure 3**). However, knockdown of duTRIM29 was significantly upregulated the expression of IFN-β, IRF7, Mx, and IL-6 mediated by duRIG-I (**Figure 4**). Thus, these results demonstrate that duTRIM29 inhibits duRIG-I-mediated IFN-β production.

### DuTRIM29 inhibits IFN-β and immune-related gene expression mediated by duMAVS

**Figure 5:**
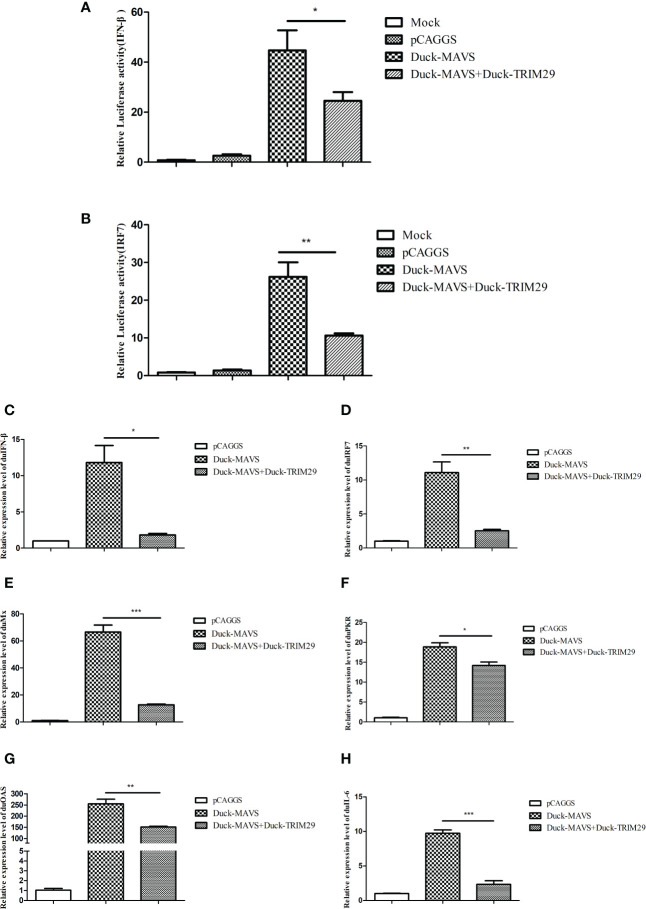
DuTRIM29 inhibits IFN-β and immune-related gene expression mediated by duMAVS. **(A, B)** DuTRIM29-HA, duMAVS-V5, or pCAGGS together with IFN-β-Luc **(A)**, IRF7-Luc **(B)** and pRL-TK were transfected into DEF cells. IFN-β and IRF7 promoter activity were measured after 24 h. **(C–H)** DuMAVS-V5, duTRIM29-HA, or pCAGGS was transfected into DEF cells. After 24 h, the relative expression of IFN-β **(C)**, IRF7 **(D)**, Mx **(E)**, PKR **(F)**, OAS**(G)**, and IL-6**(H)** was measured by qRT-PCR. Statistics were analyzed *via* unpaired two-tailed Student’s t test: *P < 0.05; **P < 0.01; ***P < 0.001.

**Figure 6:**
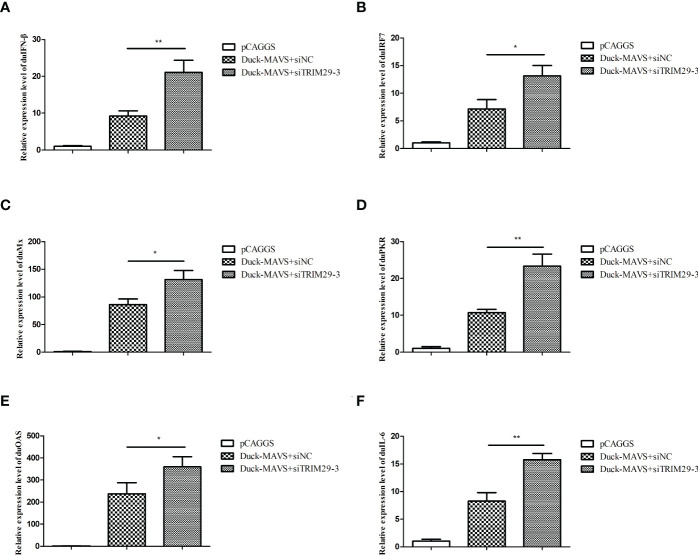
Knockdown of duTRIM29 promotes IFN-β and immune-related gene expression mediated by duMAVS. DEF cells were cotransfected with duMAVS-V5, siTRIM29-3, or siNC. After 24 h, the relative expression of IFN-β **(A)**, IRF7 **(B)**, Mx **(C)**, PKR **(D)**, OAS **(E)**, and IL-6 **(F)** was measured by qRT-PCR. Statistics were analyzed *via* unpaired two-tailed Student’s t test: *P < 0.05; **P < 0.01.

In mammals, TRIM29 plays a negative role in IFN-I production in the MAVS-mediated innate immune signaling pathway. To investigate whether duTRIM29 regulates IFN-β and immune-related gene expression mediated by duMAVS, DEFs were transfected with duTRIM29-HA or siTRIM29-3 and duMAVS-V5 for qRT-PCR and luciferase reporter assays. As shown in **Figure 4**, overexpression of duTRIM29 significantly inhibited avian IFN-β and IRF7 promoter activation and downregulated the expression of IFN-β, IRF7, Mx, PKR, OAS and IL-6 mediated by duMAVS (**Figure 5**). In addition, knockdown of duTRIM29 was significantly upregulated the expression of IFN-β, IRF7, Mx, PKR, OAS and IL-6 mediated by duMAVS (**Figure 6**). Therefore, our results show that duTRIM29 inhibits IFN-β and immune-related gene expression mediated by duMAVS.

### DuTRIM29 interacts with duMAVS

**Figure 7:**
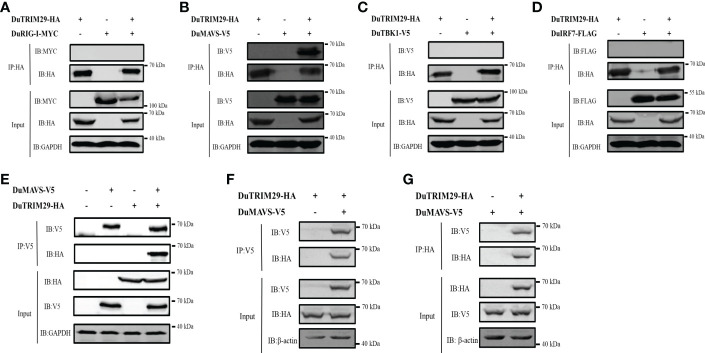
The interaction of duTRIM29 and key proteins of the duRIG-I signaling pathway. **(A–D)** HEK293T cells were transfected with duTRIM29-HA and duRIG-I-MYC **(A)**, duMAVS-V5 **(B)**, duTBK1-V5 **(C)** and duIRF7-FLAG **(D)**. After 24 h, the cellular lysates were incubated with anti-HA beads. The proteins were detected by immunoblotting. **(E)** DuTRIM29-HA and duMAVS-V5 were transfected into HEK293T cells. The cellular lysates were incubated with anti-V5 beads after 24 h, and the proteins were detected by immunoblotting. **(F, G)** DuTRIM29-HA and duMAVS-V5 were transfected into DEF cells. The cellular lysates were incubated with anti-V5 beads or anti-HA beads after 24 h, and the proteins were detected by immunoblotting.

To determine whether duTRIM29 interacts with key proteins of the duck RIG-I signaling pathway, HEK293T cells were cotransfected with duTRIM29-HA and duRIG-I-MYC, duMAVS-V5, duTBK1-V5 or duIRF7-FLAG expression plasmids. Then, we performed a Co-IP assay to detect the interacting protein of duTRIM29. The cellular lysates were incubated with anti-HA beads, and duMAVS was detected by immunoblotting using the anti-V5 antibody in immunoprecipitated protein complexes (**Figures 7A–D**). The results indicated that duTRIM29 interacted with duMAVS, but not with duRIG-I, duTBK1 or duIRF7. In addition, HEK293T cells were cotransfected with duTRIM29-HA and duMAVS-V5, and the cellular lysates were incubated with anti-V5 beads. It was confirmed that duTRIM29-HA was pulled down with duMAVS-V5 (**Figure 7E**). It was further verified the interaction between duTRIM29 and duMAVS in DEF cells (**Figures 7F–G**). Taken together, we conclude that duTRIM29 interacts with duMAVS, not with duRIG-I, duTBK1 and duIRF7.

### DuTRIM29 colocalizes with duMAVS in the cytoplasm

**Figure 8:**
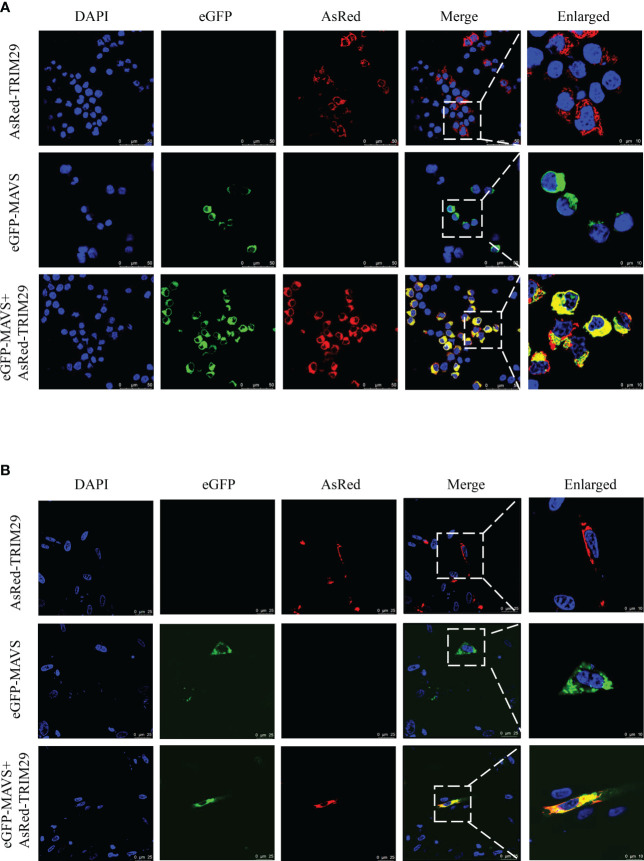
DuTRIM29 colocalizes with duMAVS in HEK293T cells and DEF cells. **(A, B)** HEK293T cells **(A)** and DEF cells **(B)** were grown on coverslips in 24-well plates and transfected with duTRIM29-AsRed and duMAVS-eGFP. After 24 h, the cells were visualized by the confocal laser scanning microscopy.

To determine whether duTRIM29 colocalizes with duMAVS by confocal microscopy, duMAVS-eGFP and duTRIM29-AsRed were cotransfected into HEK293T cells and DEF cells, respectively. When duMAVS-eGFP or duTRIM29-AsRed was expressed alone in HEK293T cells, duMAVS-eGFP or duTRIM29-AsRed was mainly located in the cytoplasm. When duMAVS-eGFP and duTRIM29-AsRed were coexpressed, duMAVS-eGFP colocalized with duTRIM29-AsRed in the cytoplasm of HEK293T cells (**Figure 8A**). Similarly, the colocalization of duTRIM29 and duMAVS in DEFs was also confirmed (**Figures 8B**, **S1**). The results indicate that duTRIM29 colocalizes with duMAVS in the cytoplasm.

### DuTRIM29 interacts with duMAVS *via* its C-terminal domains

**Figure 9:**
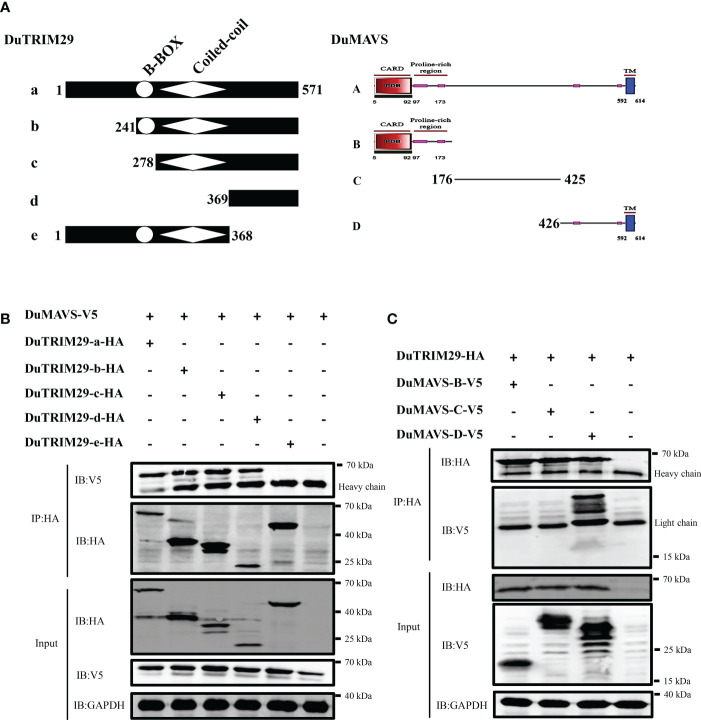
DuTRIM29 interacts with duMAVS *via* its C-terminal domains. **(A)** Schematic structures of duTRIM29 and duMAVS. **(B)** Full-length and truncation mutants of duTRIM29-HA were cotransfected with duMAVS-V5 into HEK293T cells. The cellular lysates were incubated with anti-HA beads after 24 h, and the proteins were detected by immunoblotting. **(C)** Full-length duTRIM29-HA was cotransfected with truncation mutants of duMAVS-V5 into HEK293T cells. The cellular lysates were incubated with anti-HA beads after 24 h, and the proteins were detected by immunoblotting. a: (1-571 aa), b: (241-571 aa), c: (278-571 aa), d: (369-571 aa) and e: (1-368 aa); A: (1-614 aa), B: (1-175 aa), C: (176-425 aa) and D: (426-614 aa).

To further map the interaction domain between duTRIM29 and duMAVS (**Figure 9A**), duMAVS-V5 and full-length and truncation mutants of duTRIM29 with HA-tag were cotransfected into HEK293T cells. Co-IP results showed that both the full-length and C-terminal domains (amino acids 369-571) of duTRIM29 interacted with duMAVS (**Figure 9B**). Next, duTRIM29-HA and truncation mutants of duMAVS with V5-tag were cotransfected into HEK293T cells. Co-IP results indicated that the C-terminal domain (amino acids 426-614) of duMAVS bound to duTRIM29 (**Figure 9C**). Thus, our results demonstrate that duTRIM29 interacts with duMAVS *via* its C-terminal domains.

### DuTRIM29 promotes K29-linked polyubiquitination and degradation of duMAVS to inhibit IFN-β production

**Figure 10:**
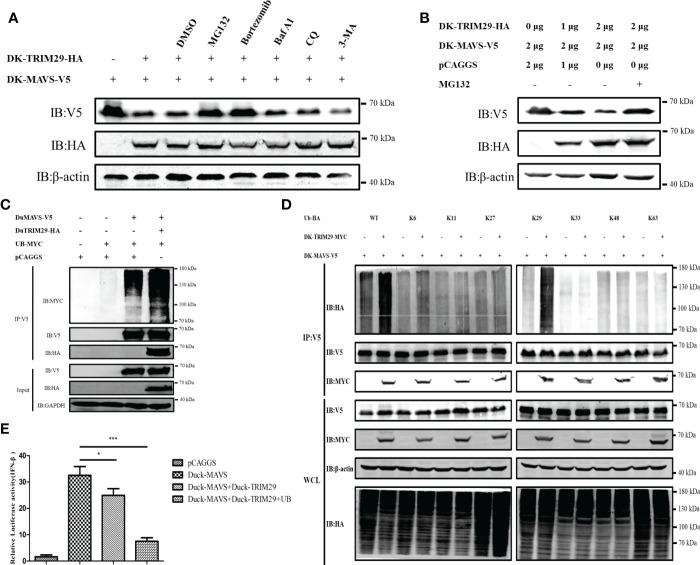
DuTRIM29 promotes K29-linked polyubiquitination and degradation of duMAVS to inhibit IFN-β production mediated by duMAVS. **(A)** DuTRIM29-MYC, duMAVS-V5 or pCAGGS was transfected into HEK293T cells. After 24 h, HEK293T cells were treated with proteasome inhibitors (10 μM MG132 and 100 nM bortezomib) and autophagy inhibitors (0.4 μM Baf A1, 50 μM CQ and 5mM 3-MA) for 8 h, and the proteins were detected by immunoblotting. **(B)** DuTRIM29-MYC, duMAVS-V5 or pCAGGS was transfected into HEK293T cells. After 24 h, HEK293T cells were treated with 10 μM MG132 for 8 h, and the proteins were detected by immunoblotting. **(C)** DuTRIM29-HA, duMAVS-V5, UB-MYC or pCAGGS was transfected into HEK293T cells. The cellular lysates were incubated with anti-V5 beads after 24 h, and the proteins were detected by immunoblotting. **(D)** DuTRIM29-MYC, duMAVS-V5, UB-HA (WT, K6, K11, K27, K29, K33, K48 and K63) or pCAGGS was transfected into HEK293T cells. The cellular lysates were incubated with anti-V5 beads after 24 h, and the proteins were detected by immunoblotting. **(E)** DuTRIM29-HA, duMAVS-V5, UB-MYC or pCAGGS together with IFN-β-Luc and pRL-TK were transfected into DEF cells. IFN-β promoter activity was measured after 24 h. Statistics were analyzed *via* unpaired two-tailed Student’s t test: *P < 0.05; ***P < 0.001. .

To confirm whether duTRIM29 affects the expression of duMAVS, HEK293T cells were treated with proteasome inhibitors (MG132 and bortezomib) and autophagy inhibitors (Baf A1, CQ and 3-MA) after overexpression of duTRIM29 and duMAVS. The results showed that overexpression of duTRIM29 accelerated the degradation of duMAVS, while the treatment of MG132 and bortezomib rescue the expression of duMAVS. However, Baf A1, CQ and 3-MA do not rescue the expression of duMAVS (**Figures 10A, B**). Thus, duTRIM29 degrades duMAVS by ubiquitin-proteasome system. To study whether duTRIM29 ubiquitinylates duMAVS, duTRIM29-HA, duMAVS-V5 and UB-MYC were cotransfected into HEK293T cells. Co-IP results showed that duTRIM29 significantly enhanced the polyubiquitination of duMAVS *via* its E3 ubiquitin ligase activity (**Figure 10C**). In addition, after overexpression of duTRIM29-MYC, duMAVS-V5 and WT or mutated Ub-HA in HEK293T cells, we found that duTRIM29 promoted the K29-linked polyubiquitination of duMAVS (**Figure 10D**). To determine whether duTRIM29 ubiquitylates duMAVS to inhibit IFN-β production mediated by duMAVS, DEF cells were cotransfected with duTRIM29-HA, duMAVS-V5 and UB-MYC together with pGL3-IFN-β-Luc and PRL-TK. As shown in **Figure 8B**, duTRIM29 significantly inhibited duMAVS-mediated IFN-β promoter activity (**Figure 10E**). Therefore, these results indicate that duTRIM29 promotes K29-linked polyubiquitination and degradation of duMAVS, leading to inhibit IFN-β production mediated by duMAVS.

## Discussion

TRIM family proteins comprise more than 80 members in humans and share a similar characteristic structure, which contains a RING domain, one or two B-box domains, a coiled-coil domain and a variable C-terminal domain (22, 23). According to the differences in C-terminal domains, TRIM proteins are divided into 11 subtypes (24). TRIM proteins are ubiquitous in vertebrate and invertebrate species and have been implicated in many cellular processes, including autophagy, carcinogenesis, pyroptosis, antiviral innate immunity and adaptive immunity (25–27). In mammals, TRIM29 encompasses a B-box and a coiled-coil domain, but not a RING finger domain (28). Specifically, TRIM29 has a B-box domain that also possesses E3 ubiquitin ligase activity (29). In this study, we first identified TRIM29 in ducks, which contained a 1716 bp ORF encoding 571 amino acids. Similar to mammalian TRIM29, duTRIM29 was composed of a B-box domain and a coiled-coil domain. Thus, duTRIM29 also lacked the conserved RING domain. We also found that duTRIM29 was highly homologous to goose, chicken and other birds (89.3%-98.6%), moderately homologous to human, mouse and other mammals (62.2%-64.7%), and weakly homologous to tibetan frog (46.4%) and african clawed frog (44.6%). Therefore, these results indicate that TRIM29 is a conserved gene in different species, and may have a similar function.

The TRIM protein family has been shown to play critical roles in innate immune signaling pathways. Several TRIM proteins have been identified that positively or negatively regulate the RLR pathway by targeting PRRs or downstream adaptor proteins in mammals. For instance, TRIM4 and TRIM25 positively regulated IFN-I production by targeting RIG-I (15, 30). TRIM14, TRIM21, TRIM31 and TRIM44 interact with MAVS to promote innate immune responses (31–34). TRIM11 and TRIM27 target TBK1 to inhibit IFN-β production (35, 36). TRIM28 downregulates IFN-I expression by interacting with IRF7 (37). In mammals, TRIM29 plays a critical role in suppressing IFN-I and proinflammatory cytokine production by interacting with NEMO, MAVS and STING (8–11). A recent study demonstrated that duTRIM32 interacted with STING to promote STING-mediated IFN-β expression in ducks (7). In this study, we found that duTRIM29 interacted with duMAVS and inhibited IFN-β and immune-related gene (IRF7, IL-6, OAS, PKR and Mx) expression mediated by duMAVS. Thus, our results demonstrate that duTRIM29 negatively regulates the IFN-I signaling pathway by targeting MAVS in ducks.

Ubiquitination is one of the major conventional protein posttranslational modifications that alters the stability, subcellular localization and protein recruitment of target proteins (38). TRIM proteins are a class of E3 ubiquitin ligases involved in ubiquitination modification (39). In mammals, TRIM65 catalyzes K63-linked polyubiquitination of MDA5 and activates the MDA5-mediated signaling pathway (40). TRIM7 promoted K48-linked polyubiquitination of STING to induce protein degradation by proteasome (41). However, STING is activated by TRIM56-mediated ubiquitination (42). TRIM35 promoted RIG-I pathway mediated type I IFN production by catalyzing K63-linked polyubiquitination of TRAF3 (17). TRIM21 and TRIM26 limit IFN-I production by targeting IRF3 for ubiquitination (43, 44). In human, TRIM14, TRIM29 and TRIM44 as TRIM proteins lacking the RING domain regulate innate immunity through different mechanisms. TRIM14-MAVS interaction is essential for mitochondrial localization of WHIP-TRIM14-PPP6C complexes to promotes RIG-I-mediated innate immunity (45). TRIM44 inhibits the K48-linked ubiquitination and enhances the stability of MAVS to positively regulate antiviral immune (34). TRIM29 antagonizes host immune responses by mediating the ubiquitination and degradation of STING (K48-linkage), NEMO (K48-linkage), MAVS (K11-linkage) and TAB2 (K48-linkage) (8–11, 29). In this study, duTRIM29 induced K29-linked polyubiquitination and degradation of duMAVS to inhibit IFN-β production mediated by duMAVS. In contrast to human TRIM29, duTRIM29 targeted duMAVS for K29-linked polyubiquitination, whereas human TRIM29 mediates MAVS for K11-linked polyubiquitination. So, duck TRIM29 and human TRIM29 regulate innate immune pathway through a different strategy. Overall, our results suggest that duTRIM29 mediates K29-linked polyubiquitination and degradation of duMAVS to negatively regulate IFN-I production in the duck RIG-I signaling pathway.

In summary, duck TRIM29 was identified for the first time. DuTRIM29 interacted with duMAVS and inhibited IFN-β and immune-related gene expression mediated by duMAVS. Furthermore, duTRIM29 induced K29-linked polyubiquitination and degradation of duMAVS to negatively regulate the MAVS-mediated signaling pathway in ducks. Thus, we identified duTRIM29 as an important negative regulator of IFN-β production in the RIG-I signaling pathway in ducks. These data will contribute to a better understanding of the molecular mechanism regulating the innate immune response by TRIM proteins in ducks.

## Data availability statement

The datasets presented in this study can be found in online repositories. The names of the repository/repositories and accession number(s) can be found in the article/**Supplementary Material**.

## Author contributions

WL and PJ designed this study and performed the main experiments. YS, YDu, ZHu, MZ, ZC, ZHe, YDi, JZ and LZ performed the experiments. WL and PJ drafted the manuscript. HS participated in writing the discussion. All authors have read and approved the final manuscript. All authors contributed to the article and approved the submitted version.
