# Supplementary material for: Duck TRIM29 negatively regulates type I IFN production by targeting MAVS
Source: Front Immunol. 2023 Jan 6;13:1016214. doi: 10.3389/fimmu.2022.1016214 (PMC9853200; doi:10.3389/fimmu.2022.1016214)
Supplement: Supplementary file 1 [file DataSheet_1.docx]

**Figure Legends**

**Figure S1. DuTRIM29 colocalizes with duMAVS in DEF cells.**

DEF cells were grown on coverslips in 24-well plates and transfected with duTRIM29-AsRed and duMAVS-eGFP. After 24 h, the cells were visualized by the confocal laser scanning microscopy.

**The sequence of shelduck tripartite motif containing 29 (TRIM29)**

Organism: Anas zonorhyncha (Shelduck)

Gene symbol: TRIM29

Gene description: Tripartite motif containing 29

GenBank Numbers: OP822045

Shelduck tripartite motif containing 29 (TRIM29) nucleotide sequence:

ATGGAAACGGGGAGCGCAGCAAGGACAAATGGTACCGCCGGCAAGCCAGAGGATGTGAAGAGCCCGTCCGCCCCCAAAAAAGATGAAGAAGTGAAGAAGAACTCGAACCCTGGTGGAGGGGAGAAGGAGCCAATGAAGGGCACTGGTGGTACTTCTCTGGAGACGGGGCAAATCAAGAGCTCCCTCTTCTCTGGGAGTGACTGGAAGAGGCCCATCATTCAGTTTGTGGAGTCGTCCGATGAGAAGAGATCGACCTACTTCAGCATGGACTCGGCAGACTCGAAGAAGATGCAGTACAGCAGCGGACAGATAGGAGACATGAGGAGACCCCCCCTCTCCTTCGCAGATAAAGGCGACCTCAGGAAGTCCCTCTTCTCCTTGGATTCCAAAAAGAGCTTCCTGCCTAACGAAGGGGAAGGGAGGAAGCCGCTGTTCTCCGGCGGGCAGATGGGGGACATGAAGAAGTCTTCCCTGCCTCTGGTGGAGACCGGGGACCTGAGAAGAGCCACCTTCAACAAGGTGCCCGACAGAGCAGCCGGGTCGCGGCCCAGGGTGAAGCTGGAGGATGTGCTGTGCGATTCCTGCATCGACAACAAGCAAAAGGCCGTGAAGTCCTGCTTGGTGTGCCAGGCTTCCTTCTGCGAGCTGCACCTCAAGCCCCACCTGGAGGGAGCGGCTTTCCGGGACCACCAGCTCCTGGACCCCATCAGGGACTTTGAAGCAAGAAAATGCCCTGTGCATGGGAAGACCATGGAGCTGTTCTGTCAGACAGACCAGATGTGCATCTGCTACCTCTGCATGTTCCAGGAGCACAAGAACCACAGCACGGTGACGGTGGAGATCGAGAAAGCGGGTAAAGAGGCTGAGCTTTCACTGCAGAAAGAGCAACTGCAGCTGAAGATCATCGAGGTAGAGGATGAAATGGACAAGTGGCAGAAGGAGAGGGACCGCATCAAGAACTACACCACCAACGAGAAAGCCACAGTAGACCAGCATTTCAAAGAGCTGATCCGTGACCTGGAGAGGCAGAGGGATGAAGTGAAGGCTGCCCTGGACCAGAGGGAAAAGATTGCATCAGAGAACGTGAAGGAGATTGTGGATGAGCTGGAAGAGAGGGCGAAGCTGCTGCGGGAGGACAAGGAGAACAGGGAGCAGATCCACCAGATCAGTGACTCCGTGCTCTTCCTCCAGGAGTTTGGGGCTTTGATGCGGAACTATGTCCCCCCTCCATCCCTCCCGACATACAGCGTGCTGCTTGAAGGGGAGAGCATGAGCCCCTCTATGGGGCTGCTCAGAGATGACCTCCTCAACGTCTGCATGAGGCACGTGGAGAAGATCTGCAAGGCAGACCTGGGCCGCAACTTCATCGAGAGGAACCACATGGAGAACGGCGACCACCGGTTCATGATGAACAACTACGAGTGGAACCAACCCGACAACTTGAAGAGATTTTCCATGTTCCTGTCTCCCAAAGTAGGCAATGGCACTAAGCTGCCTTTTCAGTTCTCCTCGGTGGGACAGAATCCGCCCGGTGACTTCAGCAAACAGTCTGATGGGAGCCTCTTCACTAAGACCGCTTATCCCTCGATAGTGAGACATCAGTCTGCAAAGGTGACGCCACAGACGTGGAAATCCTCCAAGCAGTCTGTGTTGTCACATTACCGCCCCTTTTACGTCAACAAAGGCAATGGAGCCACCTCCAACGAGGCACCTTGA

Shelduck tripartite motif containing 29 (TRIM29) amino acid sequence:

METGSAARTNGTAGKPEDVKSPSAPKKDEEVKKNSNPGGGEKEPMKGTGGTSLETGQIKSSLFSGSDWKRPIIQFVESSDEKRSTYFSMDSADSKKMQYSSGQIGDMRRPPLSFADKGDLRKSLFSLDSKKSFLPNEGEGRKPLFSGGQMGDMKKSSLPLVETGDLRRATFNKVPDRAAGSRPRVKLEDVLCDSCIDNKQKAVKSCLVCQASFCELHLKPHLEGAAFRDHQLLDPIRDFEARKCPVHGKTMELFCQTDQMCICYLCMFQEHKNHSTVTVEIEKAGKEAELSLQKEQLQLKIIEVEDEMDKWQKERDRIKNYTTNEKATVDQHFKELIRDLERQRDEVKAALDQREKIASENVKEIVDELEERAKLLREDKENREQIHQISDSVLFLQEFGALMRNYVPPPSLPTYSVLLEGESMSPSMGLLRDDLLNVCMRHVEKICKADLGRNFIERNHMENGDHRFMMNNYEWNQPDNLKRFSMFLSPKVGNGTKLPFQFSSVGQNPPGDFSKQSDGSLFTKTAYPSIVRHQSAKVTPQTWKSSKQSVLSHYRPFYVNKGNGATSNEAP.
