# Supplementary figures and images for: Duck TRIM29 negatively regulates type I IFN production by targeting MAVS
Source: Front Immunol. 2023 Jan 6;13:1016214. doi: 10.3389/fimmu.2022.1016214 (PMC9853200; doi:10.3389/fimmu.2022.1016214)

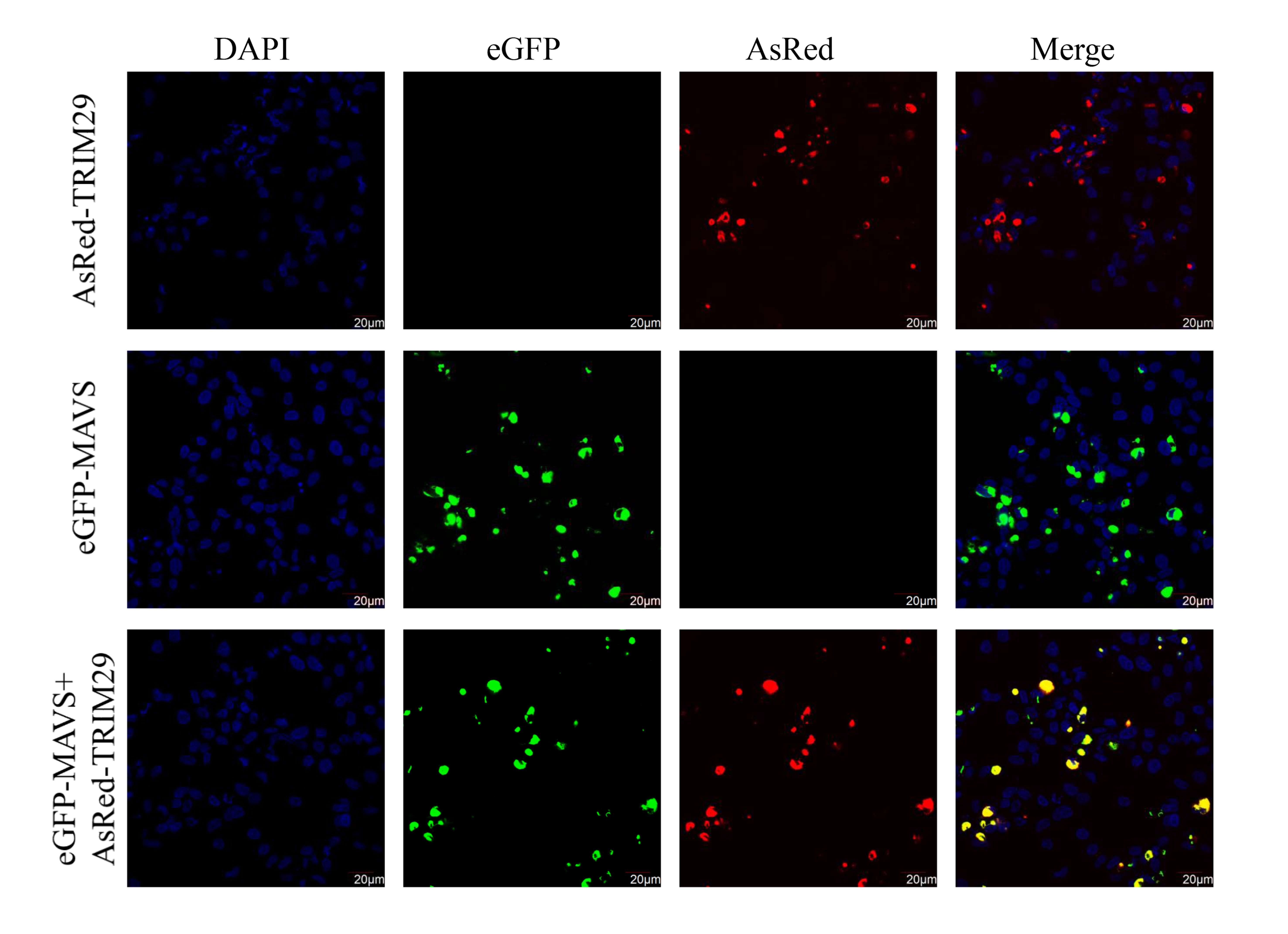

Supplement: Supplementary file 2 [file Image_1.jpeg]
